# An Analytic Nuclear Energy Gradient for Multicomponent CCSD

**DOI:** 10.1021/acs.jctc.6c01090

**Published:** 2026-08-23

**Authors:** Gabrielle B. Tucker, Kurt R. Brorsen

**Affiliations:** Department of Chemistry, 14716 University of Missouri, Columbia, Missouri 65211, United States

## Abstract

An analytic nuclear energy gradient for multicomponent coupled-cluster theory with single and double excitations (CCSD) is introduced along with the corresponding Z-vector equations, assuming that the wave function of the quantum nuclei is a Hartree product and no proton–proton double excitation operators are included in the cluster operator. The analytic gradient is used to optimize molecular geometries to determine vibrationally averaged bond distances and angles, from which rotational constants are calculated. The Z-vector methodology is used to calculate relaxed protonic densities and dipole moments at vibrationally averaged geometries. Due to the essential role that analytic energy gradients play in electronic structure theory, we expect the analytic nuclear energy gradient to enable many applications for including nuclear quantum effects in coupled-cluster calculations.

## Graphical abstract

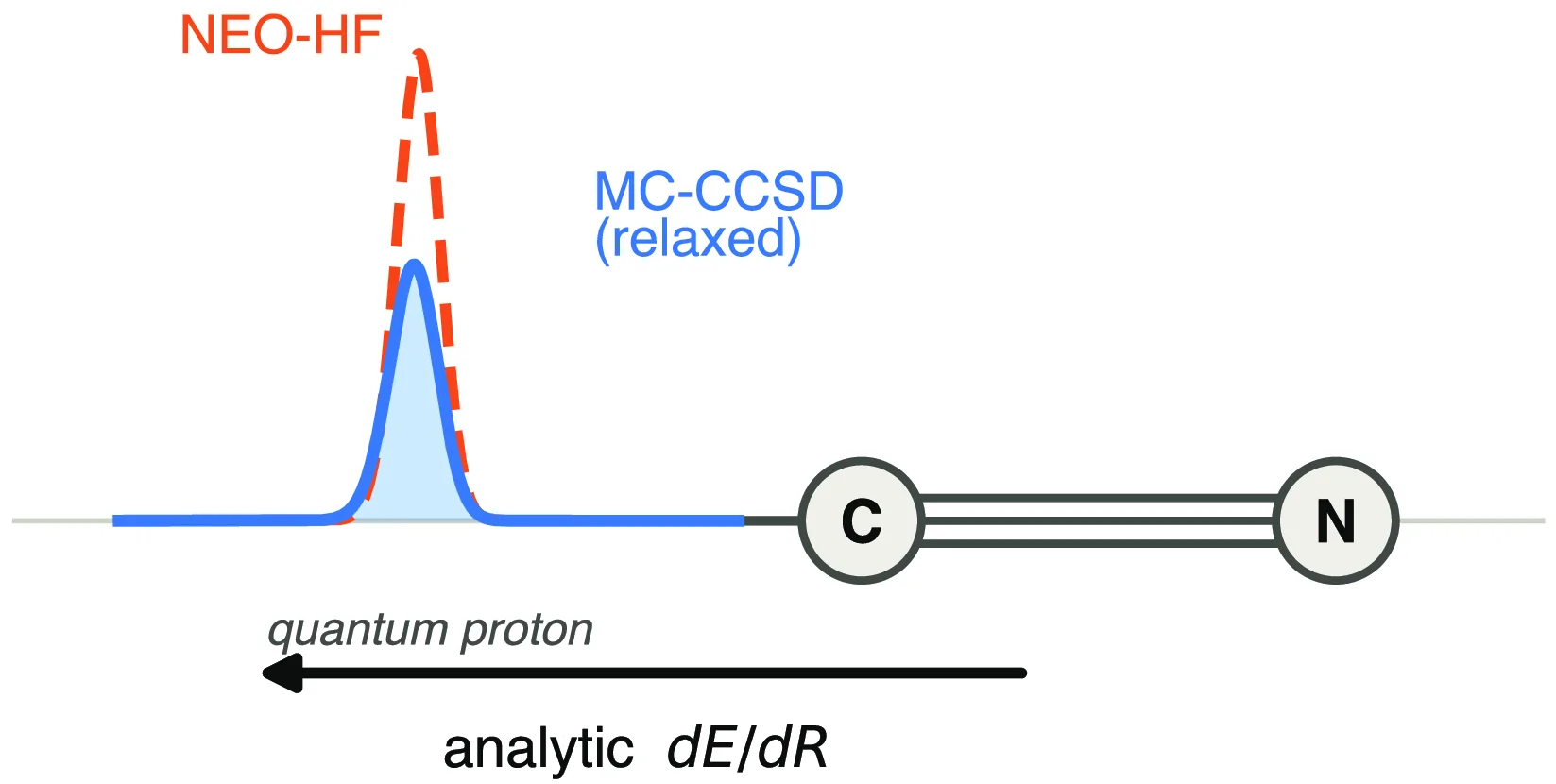

## 1 Introduction

Analytic energy gradients are an essential part of many mature *ab initio* electronic structure theory methods.^1^ Analytic nuclear energy gradients allow for efficient geometry optimizations to be performed, which is important, as the number of energy evaluations for numerical nuclear energy gradients scales roughly as 6*N*, where *N* is the number of atoms in the system. More generally, analytic energy gradients enable the efficient calculation of molecular properties such as polarizabilities, spin–spin coupling constants, and harmonic vibrational frequencies among others.^2^

Analytic energy gradients for nonvariational electronic structure theory methods are more complicated than for variational methods due to the need to include the orbital response even for first derivatives. Implementations of coupled-cluster (CC) energy gradients were first introduced in the 1980s,^3–7^ and the Lagrangian framework is now well established as the preferred means to evaluate CC energy gradients and molecular properties.^8–10^ Due to their high accuracy with acceptable computational scaling, for single-reference systems, CC methods are commonly the methods of choice for computing molecular properties and are often used for benchmarking.^2^

One important branch of coupled-cluster theory methods that currently lacks analytic energy gradients is multicomponent CC theory. Multicomponent methods are a class of electronic structure theory methods that include nuclear quantum effects in quantum chemistry calculations by treating multiple types of particles quantum mechanically on equal footing.^11–14^ In most applications of multicomponent methods, these particles are electrons and (select) protons, but multicomponent methods can treat more exotic particles quantum mechanically as well.^15–19^ In the field of multicomponent methods, the standard methods of quantum chemistry are normally referred to as single-component methods, as a single component of the system (electrons) is treated quantum mechanically.

Multicomponent methods are appealing for the calculation of molecular properties due to their potential energy surfaces (PESs) being intrinsically vibrationally averaged, which allows for the calculation of changes in molecular properties due to vibrational motion from a single geometry optimization.^20^ Additionally, the quantum nuclear masses are input parameters in multicomponent calculations, which makes calculating changes in molecular properties due to isotopic substitution trivial.^21–23^

Multicomponent CC methods were introduced in 2003 by Nakai and coworkers, who formulated multicomponent coupled-cluster theory at the CC doubles and Brueckner doubles levels.^24^ A multicomponent CC ansatz with connected cross-particle excitation operators was later formulated by Chakraborty and coworkers for electron-positron and electron–hole interactions.^15^ In that work, the amplitude equations were given as Baker–Campbell–Hausdorff operator expansions and the method was applied to biexcitonic systems. The first complete set of programmable multicomponent CCSD energy and amplitude equations for quantum nuclei, with an implementation and benchmarks for molecular systems, was introduced by Pavošević and coworkers in 2018.^25^ Since that study, numerous extensions of multicomponent CC and multicomponent many-body perturbation theory have been introduced^18,26–33^ with multicomponent CCSD with perturbative triple excitations (CCSD­(T)) being called the “gold standard” of multicomponent *ab initio* methods.^28,31^

In multicomponent methods, the quantum nuclei no longer have well-defined positions in space like the classical nuclei. While there exist different definitions of the multicomponent PES, the most widely used definition requires that the positions of the nuclear basis functions be optimized for a fixed set of classical nuclear coordinates.^34^ Thus, even for an energy calculation, multicomponent methods require a geometry optimization or other specialized methodology.^35^ For calculations with a single quantum proton, it is feasible to perform this optimization numerically, but recent multicomponent CCSD calculations have been performed with up to nine quantum nuclei,^36^ which can make the optimization computationally impractical. Therefore, an analytic nuclear energy gradient is even more desirable for multicomponent methods than for single-component methods.

At present, analytic nuclear energy gradients for multicomponent methods exist for multicomponent Hartree–Fock (HF),^37^ density functional theory (DFT),^20,38^ and the complete active space self-consistent field (CASSCF) method.^37^ In this study, we derive and implement the analytic multicomponent CCSD nuclear energy gradient. Using the analytic nuclear energy gradient, we perform geometry optimizations to compute vibrationally averaged minimum-energy geometries on the multicomponent PES. We also use a small modification of the new multicomponent CCSD analytic energy gradient code to compute vibrationally averaged dipole moments at these minima.

The relaxed density matrices constructed for the analytic gradient also give the CC response density of the quantum nuclei, which, as shown below, improves the vibrationally averaged geometries and rotational constants. An analytic first derivative is furthermore a prerequisite for a seminumerical Hessian, which would make multicomponent CCSD harmonic frequencies and infrared intensities accessible for systems in which nuclear quantum effects are important. Given the importance of analytic energy gradients in single-component quantum chemistry, we expect the multicomponent CCSD analytic energy gradient to enable more diverse applications of multicomponent CCSD.

## 2 Theory

### 2.1 Multicomponent Coupled-Cluster Theory

In the following, we adopt the convention that occupied, virtual, and arbitrary electronic orbitals are denoted by *i*, *j*, *k*,...; *a*, *b*, *c*,...; and *p*, *q*, *r*,...; respectively. For simplicity, we assume all quantum nuclei are protons. Protonic orbitals are then denoted identically as the electronic orbitals, but with capital letters. We label the electronic subspace by e and the protonic subspace of nucleus α by p_α_. We emphasize that the proton label, p, is always written in roman text, while the electronic orbital label, *p*, is italicized.

In the following, we derive an analytic nuclear energy gradient for *N* distinguishable quantum nuclei. We treat the nuclei as distinguishable as exchange effects between identical nuclei have been shown to be small for multicomponent many-body methods due to the nuclei remaining well localized compared to the electrons for most chemical systems of interest.^39^ With this assumption, the multicomponent normal-ordered Hamiltonian is

$${Ĥ}_{N}=\underset{pq}{\sum }F_{p}^{q}{â}_{q}^{p}+\frac{1}{4}\underset{pqrs}{\sum }{\mathrm{g̅}}_{rs}^{pq}{â}_{pq}^{rs}+\underset{\alpha }{\sum }\underset{PQ\in \alpha }{\sum }F_{P}^{Q}{â}_{Q}^{P}-\underset{\alpha }{\sum }\underset{\begin{array}{c} pq\\ \begin{array}{c} PQ\in \alpha \end{array} \end{array}}{\sum }g_{qQ}^{pP}{â}_{pP}^{qQ}+\underset{\alpha \neq \beta }{\sum }\underset{\begin{array}{l} PQ\in \alpha \\ RS\in \beta \end{array}}{\sum }g_{QS}^{PR}{â}_{PR}^{QS}$$ 1

where *F* is the electronic or protonic Fock matrix element, $g_{QS}^{PR}=\left\langle QS\right|PR\rangle$ is a two-particle Coulomb integral, ${\mathrm{g̅}}_{rs}^{pq}=\left\langle rs\right|\left|pq\right\rangle$ is an antisymmetrized two-electron integral, ${â}_{pq...PQ...}^{rs...RS...}=\left\{{â}_{r}^{†}{â}_{s}^{†}...{â}_{R}^{†}{â}_{S}^{†}...{â}_{q}{â}_{p}...{â}_{Q}{â}_{P}...\right\}$ is an excitation operator where *â*^†^ and *â* are creation and annihilation operators, respectively, and the sum over α ≠ β occurs only over unique nuclear pairs. Electron–proton Coulomb integrals are defined analogously. We note that we do not use the Einstein summation convention and keep all summations explicit to reduce any confusion from the multiple proton indices. The multicomponent HF reference wave function is written as

2 $$\left|0\right\rangle =\left|0^{e}\right\rangle ⊗\underset{\alpha }{\prod }\left|0^{p_{\alpha }}\right\rangle$$

where |0^e^⟩ is an electronic Slater determinant and $\left|0^{p_{\alpha }}\right\rangle$ is the occupied HF orbital of proton α.

The reference is the multicomponent HF wave function with Fock operators defined using the Setup A convention of Goudy and coworkers.^31^ In this convention, the Fock operator for quantum nucleus α is

3 $${\mathrm{F̂}}^{p_{\alpha }}={ĥ}^{p_{\alpha }}+{{\mathrm{v̂}}^{\mathrm{HF}}}_{\mathrm{ep},p_{\alpha }}+\underset{\beta \neq \alpha }{\sum }{{\mathrm{v̂}}^{\mathrm{HF}}}_{p_{\beta }p_{\alpha }}$$

The multicomponent CCSD wave function is defined as |Ψ^MC–CCSD^⟩ = *e* ^*T̂*^|0⟩, where the cluster operator is defined as $\mathrm{T̂}={\mathrm{T̂}}_{1}^{e}+{\mathrm{T̂}}_{2}^{\mathrm{ee}}+\underset{\alpha }{\sum }{\mathrm{T̂}}_{1}^{p_{\alpha }}+\underset{\alpha }{\sum }{\mathrm{T̂}}_{2}^{{\mathrm{ep}}_{\alpha }}$ and includes single and double electronic excitations, single protonic excitations, and mixed double excitations of one electron and one proton. Proton–proton correlation was shown to be over a thousand times smaller than electron–proton correlation in a recent multicomponent CC study.^31^ We therefore assume it to be negligible and do not include the mixed double excitation operator for different protons, ${\mathrm{T̂}}_{2}^{p_{\alpha }p_{\beta }}$ . We note that the neglect of ${\mathrm{T̂}}_{2}^{p_{\alpha }p_{\beta }}$ is an approximation whose validity has been established only for systems in which the quantum nuclei are well separated and weakly coupled. Systems with quantum nuclei at close distances, with strong hydrogen bonding involving quantum nuclei, or with many quantum nuclei of small mass may show significantly more substantial direct proton–proton correlation. Keeping ${\mathrm{T̂}}_{2}^{p_{\alpha }p_{\beta }}$ throughout the derivation and testing its contribution over a wider range of systems is a useful direction for future work.

Following from the definitions of the Hamiltonian, reference wave function, and cluster operator, the multicomponent CCSD correlation energy functional^40^ is defined as

4 $$L=\left\langle 0\left|\left(1+\mathrm{\Lambda ̂}\right)e^{-\mathrm{T̂}}{Ĥ}_{N}e^{\mathrm{T̂}}\right|0\right\rangle$$

where $\mathrm{\Lambda ̂}={\mathrm{\Lambda ̂}}_{1}^{e}+{\mathrm{\Lambda ̂}}_{2}^{\mathrm{ee}}+\underset{\alpha }{\sum }{\mathrm{\Lambda ̂}}_{1}^{p_{\alpha }}+\underset{\alpha }{\sum }{\mathrm{\Lambda ̂}}_{2}^{{\mathrm{ep}}_{\alpha }}$ is the Λ de-excitation operator. Because *Ĥ*_*N*_ is normal ordered, L is the correlation energy rather than the total energy. Making L stationary with respect to the T amplitudes and Λ amplitudes gives the amplitude equations. The total energy is the sum of L and the multicomponent HF energy. It can be written in terms of the one- and two-particle density matrices:

$$\begin{array}{ll} E_{\mathrm{MC}-\mathrm{CCSD}} & =\underset{pq}{\sum }\gamma_{pq}^{e}h_{pq}^{e}+\frac{1}{4}\underset{pqrs}{\sum }\Gamma_{pqrs}^{\mathrm{ee}}{\overset{¯}{g}}_{rs}^{pq}+\underset{\alpha }{\sum }\underset{PQ\in \alpha }{\sum }\gamma_{PQ}^{p_{\alpha }}h_{PQ}^{p_{\alpha }}-\underset{\alpha }{\sum }\underset{\begin{array}{c} pq\\ \begin{array}{c} PQ\in \alpha \end{array} \end{array}}{\sum }\Gamma_{pqPQ}^{{\mathrm{ep}}_{\alpha }}g_{qQ}^{pP}\\ & +\underset{\alpha \neq \beta }{\sum }\underset{\begin{array}{l} PQ\in \alpha \\ RS\in \beta \end{array}}{\sum }\Gamma_{PQRS}^{p_{\alpha }p_{\beta }}{\overset{¯}{g}}_{QS}^{PR}+V_{\mathrm{nuc}} \end{array}$$ 5

where γ^e^ and $\gamma^{p_{\alpha }}$ are the one-particle density matrices for electrons and protons, Γ^ee^ is the two-electron density matrix, $\Gamma^{p_{\alpha }p_{\beta }}$ is the proton–proton two-particle density matrix, $\Gamma^{{\mathrm{ep}}_{\alpha }}$ is the electron–proton two-particle density matrix, *h* is the one-particle Hamiltonian matrix element, and *V*_nuc_ is the classical nuclear repulsion. Although no proton–proton double excitation operators are included in the cluster operator, the proton–proton 2-PDM does not reduce to a product of the protonic 1-PDMs. It contains a connected part that is mediated by the electrons, which is included in our derivation and implementation. The remaining density matrices include both the HF reference and correlation contributions and are constructed from the converged T amplitudes and Λ amplitudes.

We direct the reader interested in more details about the multicomponent CCSD T amplitudes,^25^ Λ amplitudes,^40^ and density matrices^40^ to the relevant previous studies.

### 2.2 Multicomponent CCSD Z-Vector Equation

Analytic CCSD nuclear energy gradients require relaxed density matrices. These can be obtained by adding the Z-vector contribution that accounts for the response of the molecular orbital coefficients to the nuclear perturbation.^41^ By solving a Z-vector equation, the explicit solution of the coupled-perturbed equations for each nuclear coordinate is not needed.

In single-component CCSD gradient theory, the equation the Z-vector satisfies is commonly implemented as **A** **·** **z** = −**X**, where **A** is the orbital Hessian and **X** is the virtual-occupied block of the Lagrangian derivative with respect to orbital rotations. This is because when the orbital Hessian is symmetric, this equation is equivalent to its transpose.

In the multicomponent framework, the orbital Hessian has the block structure^34,42^

6 $$A=\left(\begin{array}{lll} A_{\mathrm{ee}} & \begin{array}{ll} A_{{\mathrm{ep}}_{1}} & ... \end{array} & A_{{\mathrm{ep}}_{N}}\\ \begin{array}{l} A_{p_{1}e}\\ \vdots \end{array} & \begin{array}{ll} A_{p_{1}p_{1}} & ...\\ \vdots & \ddots \end{array} & \begin{array}{l} A_{p_{1}p_{N}} \end{array}\\ A_{p_{N}e} & A_{p_{N}p_{1}} & A_{p_{N}p_{N}} \end{array}\right)$$

where **A**_ee_ is the electronic orbital Hessian, $A_{p_{\alpha }p_{\alpha }}$ is the protonic orbital Hessian, the off-diagonal blocks $A_{{\mathrm{ep}}_{\alpha }}$ and $A_{p_{\alpha }e}$ couple the electronic and protonic orbital rotations through the electron–proton Coulomb interaction, and the $A_{p_{\alpha }p_{\beta }}$ and $A_{p_{\beta }p_{\alpha }}$ blocks couple the protonic orbital rotations of different protons through the proton–proton Coulomb interaction. As noted by Yang and coworkers in the context of the multicomponent HF stability analysis,^42^ when using a spin-adapted formalism, the off-diagonal electron–proton coupling blocks are not transposes of each other $\left(A_{{\mathrm{ep}}_{\alpha }}\neq A_{p_{\alpha }e}^{T}\right)$ , making the full orbital Hessian asymmetric. The interested reader is directed to refs 34 and 42 for more detail about the multicomponent orbital Hessian.

This asymmetry is a consequence of the mixed spin–orbital convention: the electrons are treated in a restricted closed-shell formalism with a spatial-orbital density prefactor of two, while each protonic species is treated in an unrestricted high-spin formalism with a density prefactor of one. The electron–proton Coulomb integral itself satisfies (*ai*|*BJ*) = (*BJ*|*ai*) but the prefactors carried into $A_{{\mathrm{ep}}_{\alpha }}$ and $A_{p_{\alpha }e}$ from the closed-shell and high-spin spin summations differ, so the two off-diagonal blocks are not transposes of each other when expressed in spatial-orbital form. In a fully spin–orbital formalism the orbital Hessian would be symmetric; the asymmetry encountered here is a fingerprint of the spin–orbital reduction rather than a consequence of the underlying physics. The presence of multiple quantum protons does not alter this structure.

Following from the structure of the multicomponent CC Lagrangian, the correct Z-vector equation in the multicomponent case is the true transpose form:

7 $$A^{T}\cdot z=-X$$

This arises as the Z-vector enters the gradient through a term of the form **z** ^**T**^ **· A · U**, where **U** is the CPHF orbital response to the nuclear perturbation. Requiring the Lagrangian to be stationary with respect to **U** yields **A^T^· z = −X** rather than **A · z = −X**. In single-component theory where **A** is symmetric, the two forms are identical; in the multicomponent case, they are not.

We note that the asymmetry of the multicomponent orbital Hessian can be removed by a similarity transformation. In our convention, the spin summation gives $A_{p_{\alpha }e}=2{\left(A_{{\mathrm{ep}}_{\alpha }}\right)}^{T}$ , so the scaling matrix is $S=\mathrm{diag}\left(I_{e},\frac{1}{\sqrt{2}}I_{p_{1}},...,\frac{1}{\sqrt{2}}I_{p_{N}}\right)$ , where **I**_e_ and $I_{p_{\alpha }}$ are identity matrices on the electronic and protonic rotation spaces, and the transformed orbital Hessian *Ã* = **SAS** ^–1^ is symmetric. The transformation is compatible with the transpose form of the Z-vector equation in eq 7, which becomes **Ã**(**S** ^–1^ **z**) = −**S** ^–1^ **X**. **Ã** is also positive definite at the multicomponent HF solutions we studied, so the transformed equation can be solved with methods that require a symmetric positive definite matrix, such as the conjugate gradient method. The two routes give the same Z-vector to 5 × 10^–15^.

The solution, **z**, consists of the virtual-occupied blocks, $z_{ai}^{e}$ and $z_{AI}^{p_{\alpha }}$ , of the electronic and protonic subspaces, respectively. These Z-vector amplitudes determine the orbital-response increments Δγ^e^ and ${\Delta \gamma }^{p_{\alpha }}$ that are added to the unrelaxed one-particle density matrices (γ^unrel^). Each Δγ is a symmetric matrix whose virtual-occupied and occupied-virtual blocks are filled by the corresponding Z-vector amplitudes, with all occupied–occupied and virtual–virtual elements equal to zero. The relaxed one-particle density matrices (γ^rel^) used to evaluate the gradient are then $\gamma^{e,\mathrm{rel}}=\gamma^{e,\mathrm{unrel}}+{\Delta \gamma }^{e}$ for the electrons and are analogous for the protonic species.

The right-hand side of eq 7, ***X***, contains the virtual-occupied blocks $X_{ai}^{e}$ and $X_{AI}^{p_{\alpha }}$ , which are constructed by contracting the unrelaxed density matrices with the one- and two-particle integrals for the electronic block:

$$X_{ai}^{e}=\underset{q}{\sum }h_{aq}^{e}\gamma_{qi}^{e,\mathrm{unrel}}-\underset{q}{\sum }h_{qi}^{e}\gamma_{aq}^{e,\mathrm{unrel}}+\frac{1}{2}\underset{qrs}{\sum }{\overset{¯}{g}}_{rs}^{aq}\Gamma_{iqrs}^{ee,\mathrm{unrel}}-\frac{1}{2}\underset{qrs}{\sum }{\overset{¯}{g}}_{rs}^{iq}\Gamma_{aqrs}^{ee,\mathrm{unrel}}-\underset{\alpha }{\sum }\underset{\begin{array}{c} q\\ \begin{array}{c} PQ\in \alpha \end{array} \end{array}}{\sum }g_{qQ}^{aP}\Gamma_{qiPQ}^{ep_{\alpha },\mathrm{unrel}}+\underset{\alpha }{\sum }\underset{\begin{array}{c} q\\ \begin{array}{c} PQ\in \alpha \end{array} \end{array}}{\sum }g_{iQ}^{qP}\Gamma_{aqPQ}^{ep_{\alpha },\mathrm{unrel}}$$ 8

and for the protonic block:

$$X_{AI}^{p_{\alpha }}=\underset{Q}{\sum }h_{AQ}^{p_{\alpha }}\gamma_{QI}^{p_{\alpha },\mathrm{unrel}}-\underset{Q}{\sum }h_{QI}^{p_{\alpha }}\gamma_{AQ}^{p_{\alpha },\mathrm{unrel}}+\underset{\beta \neq \alpha }{\sum }\underset{\begin{array}{l} Q\in \alpha \\ RS\in \beta \end{array}}{\sum }g_{QS}^{AR}\Gamma_{IQRS}^{p_{\alpha }p_{\beta },\mathrm{unrel}}-\underset{\beta \neq \alpha }{\sum }\underset{\begin{array}{l} Q\in \alpha \\ RS\in \beta \end{array}}{\sum }g_{QS}^{IR}\Gamma_{AQRS}^{p_{\alpha }p_{\beta },\mathrm{unrel}}-\underset{\begin{array}{c} pq\\ \begin{array}{c} Q\in \alpha \end{array} \end{array}}{\sum }g_{qQ}^{pA}\Gamma_{pqQI}^{ep_{\alpha },\mathrm{unrel}}+\underset{\begin{array}{c} pq\\ \begin{array}{c} Q\in \alpha \end{array} \end{array}}{\sum }g_{qQ}^{pI}\Gamma_{pqAQ}^{ep_{\alpha },\mathrm{unrel}}$$ 9

These intermediates represent the derivative of the energy with respect to an electronic (κ_*ai*_) or protonic (κ_*AI*_) orbital rotation and vanish at the HF level by Brillouin’s theorem.

### 2.3 Analytic Nuclear Energy Gradient

In multicomponent methods, the total energy depends not only on the classical nuclear coordinates but also on the positions at which the protonic basis functions are centered, which are parameters of the wave function that are typically optimized either at a fixed classical geometry or alongside the classical nuclear geometry. We therefore define *R* to denote any of these coordinates, i.e., either a classical nuclear coordinate or a protonic basis-function center. The analytic gradient d*E*/d*R* is taken to include both cases. In single-component CCSD gradient theory,^4,8,43^ the analytic gradient with respect to a nuclear coordinate *R* takes the compact form

10 $$\frac{dE}{dR}=\underset{\mu \nu }{\sum }\gamma_{\mu \nu }^{e,\mathrm{rel}}\frac{\partial h_{\mu \nu }}{\partial R}+\frac{1}{4}\underset{\mu \nu \lambda \sigma }{\sum }\Gamma_{\mu \nu \lambda \sigma }^{\mathrm{ee},\mathrm{rel}}\frac{\partial {\mathrm{g̅}}_{\lambda \sigma }^{\mu \nu }}{\partial R}-\underset{\mu \nu }{\sum }W_{\mu \nu }\frac{\partial S_{\mu \nu }}{\partial R}+\frac{\partial V_{\mathrm{nuc}}}{\partial R}$$

where γ^e,rel^ and Γ^ee,rel^ are the total relaxed density matrices, which are the sum of the unrelaxed (correlated) contributions and the Z-vector orbital-response contributions, and **W** is the energy-weighted density matrix constructed from these relaxed quantities.

The two-particle density matrix separates into a connected (cumulant) part, which depends only on the T and Λ amplitudes, and a disconnected part, $\Gamma_{pqrs}^{\mathrm{dc}}=\gamma_{pr}\gamma_{qs}-\gamma_{ps}\gamma_{qr}$ , that is a quadratic function of the one-particle density matrix. In single-component CCSD gradient implementations,^4,6,43^ the disconnected part of the 2-PDM involving the product of the relaxed 1-PDMs is not assembled. Instead, the disconnected part is constructed from products of the unrelaxed 1-PDMs, with the HF density serving as the reference. While this introduces a contribution that is quadratic in the correlation density, the orbital-relaxation response of each product is linear in the corresponding density variation, so the relaxation enters through a single Z-vector contraction rather than a bilinear coupling of independent relaxations. Therefore, the quadratic Δγ^e^Δγ^e^ piece that would arise from forming γ^e,rel^γ^e,rel^ as a product is never generated.

The multicomponent CCSD gradient has the same general structure but includes additional terms from the protonic one-particle density matrix, the proton-proton twin-particle density metric, the electron-proton two-particle density matrix, the protonic core Hamiltonian, and the pronto overlap matrix:

11 $$\begin{array}{ll} \frac{dE}{dR} & =\underset{\mu \nu \in e}{\sum }\gamma_{\mu \nu }^{e,rel}\frac{\partial h_{\mu \nu }^{e}}{\partial R}+\frac{1}{4}\underset{\mu \nu \lambda \sigma \in e}{\sum }\Gamma_{\mu \nu \lambda \sigma }^{ee,rel}\frac{\partial {\mathrm{g̅}}_{\lambda \sigma }^{\mu \nu }}{\partial R}+\underset{\alpha }{\sum }\underset{\mu \nu \in \alpha }{\sum }\gamma_{\mu \nu }^{p_{\alpha },rel}\frac{\partial h_{\mu \nu }^{p_{\alpha }}}{\partial R}-\underset{\alpha }{\sum }\underset{\begin{array}{l} \mu \nu \in e\\ \lambda \sigma \in \alpha \end{array}}{\sum }\Gamma_{\mu \nu \lambda \sigma }^{ep_{\alpha },rel}\frac{\partial g_{\nu \sigma }^{\mu \lambda }}{\partial R}\\ & +\underset{\alpha \neq \beta }{\sum }\underset{\begin{array}{l} \mu \nu \in \alpha \\ \lambda \sigma \in \beta \end{array}}{\sum }\Gamma_{\mu \nu \lambda \sigma }^{p_{\alpha }p_{\beta },rel}\frac{\partial g_{\nu \sigma }^{\mu \lambda }}{\partial R}-\underset{\mu \nu \in e}{\sum }W_{\mu \nu }^{e}\frac{\partial S_{\mu \nu }^{e}}{\partial R}+\frac{\partial V_{\mathrm{nuc}}}{\partial R} \end{array}$$

where the Greek letters μ, ν, λ, and σ label electronic or protonic AOs and **W** ^e^ is the energy-weighted density matrix for the electrons:

$$W_{pq}^{e}=\underset{r\in e}{\sum }\gamma_{pr}^{e,\mathrm{rel}}h_{rq}^{e}+\frac{1}{2}\underset{rst\in e}{\sum }\Gamma_{prst}^{\mathrm{ee},\mathrm{rel}}{\mathrm{g̅}}_{qr}^{st}-\underset{\alpha }{\sum }\underset{\begin{array}{c} r\in e\\ \begin{array}{c} PQ\in \alpha \end{array} \end{array}}{\sum }\Gamma_{prPQ}^{{\mathrm{ep}}_{\alpha },\mathrm{rel}}g_{qQ}^{rP}$$ 12

The corresponding protonic energy-weighted density matrices, $W^{p_{\alpha }}$ , have an analogous structure but do not contribute to the gradient. This is exact and does not mean that the protonic orbitals are unrelaxed. The protonic basis-function centers are variational parameters that are optimized together with the classical nuclear coordinates, and the gradient components with respect to them are computed on the same footing. The protonic overlap-derivative term vanishes because all basis functions of a given quantum nucleus are centered on a single point. The protonic overlap matrix therefore does not change when that center is translated, so $\partial S_{\mu \nu }^{p_{\alpha }}/\partial R=0$ whether *R* is a classical nuclear coordinate, on which $S^{p_{\alpha }}$ does not depend, or the basis-function center of nucleus α itself, on which it depends only through a rigid translation. The relaxation of the protonic orbitals under nuclear displacement enters through the protonic blocks of the Z-vector rather than through an overlap-derivative term.

The most important difference for the multicomponent nuclear energy gradient compared to the single-component case is that there are now three disconnected 2-PDMs rather than one:

13 $$\Gamma_{pqrs}^{\mathrm{dc},\mathrm{ee}}=\gamma_{pr}^{e}\gamma_{qs}^{e}-\gamma_{ps}^{e}\gamma_{qr}^{e}$$

14 $$\Gamma_{PQRS}^{{\mathrm{dc},p}_{\alpha }p_{\beta }}=\gamma_{PQ}^{p_{\alpha }}\gamma_{RS}^{p_{\beta }}$$

15 $$\Gamma_{pqPQ}^{{\mathrm{dc},\mathrm{ep}}_{\alpha }}=\gamma_{pq}^{e}\gamma_{PQ}^{p_{\alpha }}$$

Note that $\Gamma^{{\mathrm{dc},p}_{\alpha }p_{\beta }}$ contains only the direct product $\gamma_{PQ}^{p_{\alpha }}\gamma_{RS}^{p_{\beta }}$ and no exchange term, reflecting the distinguishable nature of the quantum protons.

Following the single-component CCSD energy gradient, the gradient is split into an unrelaxed contribution and a response contribution:

16 $$\frac{dE}{dR}={\left(\frac{dE}{dR}\right)}_{\mathrm{unrelaxed}}+{\left(\frac{dE}{dR}\right)}_{\mathrm{response}}$$

The unrelaxed contribution uses the correlated density matrices, that is the full Γ^ee^ from the multicomponent CCSD T amplitudes and Λ amplitudes (including its disconnected piece built from γ^e,unrel^), $\Gamma^{{\mathrm{ep}}_{\alpha }}$ , and the full $\Gamma^{p_{\alpha }p_{\beta }}$ (including its disconnected piece built from $\gamma^{p_{\alpha },\mathrm{unrel}}$ and $\gamma^{p_{\beta },\mathrm{unrel}}$ ), contracted against the derivative integrals exactly as in single-component CCSD. The response contribution uses the Z-vector increments Δγ^e^ and Δγ^p^ along with linearized 2-PDM increments, retaining only terms first order in Δ*γ*:

17 $$\Delta \Gamma_{pqrs}^{\mathrm{dc},\mathrm{ee}}=\gamma_{pr}^{e,\mathrm{HF}}\Delta \gamma_{qs}^{e}+\Delta \gamma_{pr}^{e}\gamma_{qs}^{e,\mathrm{HF}}-\left(\gamma_{ps}^{e,\mathrm{HF}}{\Delta \gamma }_{qr}^{e}+{\Delta \gamma }_{ps}^{e}\gamma_{qr}^{e,\mathrm{HF}}\right)$$

18 $$\Delta \Gamma_{PQRS}^{{\mathrm{dc},p}_{\alpha }p_{\beta }}=\gamma_{PQ}^{p_{\alpha },\mathrm{HF}}\Delta \gamma_{RS}^{p_{\beta }}+\Delta \gamma_{PQ}^{p_{\alpha }}\gamma_{RS}^{p_{\beta },\mathrm{HF}}$$

19 $$\Delta \Gamma_{pqPQ}^{{\mathrm{dc},\mathrm{ep}}_{\alpha }}=\gamma_{pq}^{e,\mathrm{HF}}\Delta \gamma_{PQ}^{p_{\alpha }}+\Delta \gamma_{pq}^{e}\gamma_{PQ}^{p_{\alpha },\mathrm{HF}}$$

As in the single-component case, each response increment is linear in Δγ by construction, with HF densities as the constant factors.

### 2.4 Dipole Moments

In this study, we also calculated multicomponent CCSD dipole moments. The same machinery used for the analytic nuclear energy gradient yields the analytic dipole moment (and, more generally, analytic first derivatives with respect to any one-electron or one-proton external perturbation) by replacing the nuclear-coordinate derivatives of the integrals with the corresponding property-integral derivatives and dropping the overlap-derivative and nuclear-repulsion terms as one-electron property operators do not induce orbital reorthonormalization or classical nuclear motion. The Z-vector and the linearized disconnected densities are unchanged.

## 3 Methodology

### 3.1 Implementation

We have implemented the multicomponent CCSD analytic energy gradient in a new multicomponent CCSD code that uses the Yang group’s multicomponent-enabled PySCF fork.^44–46^ We used the PySCF fork to obtain converged electronic and protonic orbitals and to generate all geometry- and field-perturbed integrals. The multiproton CCSD energy expression, T amplitudes, Λ amplitudes, and 1- and 2-PDMs were generated symbolically using the program SASQ.^47^ The SASQ output is parsed and compiled at run time. All code generated by SASQ is spin-adapted by default. The rest of the needed functionality was written in our in-house code. Some of the in-house code, most significantly the Z-vector solver and gradient assembly, was written using assistance from Claude Code.

The code was tested on the HCN and (HF)_2_ systems with the aug-cc-pVDZ and aug-cc-pVTZ electronic and PB4-F1 protonic basis sets^48^ with the hydrogen nuclei treated quantum mechanically. To verify the amplitude equations, we compared to our previous multicomponent CCSD code. To verify the Λ amplitudes and density matrices, we reconstructed the energy using eq 5 and compared to the energy obtained from solving the amplitude equations. To verify the Z-vector solver, analytic nuclear energy gradient, and analytic dipole, we compared to finite differences. For the analytic energy gradient, the agreement was better than 1 × 10^–8^ a.u. for all elements for HCN and (HF)_2_ using the aug-cc-pVDZ or aug-cc-PVTZ and PB4-F1 basis sets. For the analytic dipole, the absolute agreement with the reference value was better than 5 × 10^–8^ a.u. for all elements for HCN and (HF)_2_ using the aug-cc-pVDZ or aug-cc-PVTZ and PB4-F1 basis sets. Exact values are shown in the Supporting Information. To reach this level of agreement between the analytic gradient and finite differences, we note that we needed to converge the multicomponent HF orbital gradient to 1 × 10^–10^ using the multicomponent augmented-Hessian method as DIIS stalled before reaching the required tolerance.^49^

### 3.2 Computational Details

We have performed geometry optimizations and calculated dipoles using the new multicomponent CCSD gradient code for several small molecules. For all multicomponent calculations, we treated all hydrogen nuclei quantum mechanically and used the PB4-F1 protonic basis set for the quantum nuclei.^48^ While there has been much recent discussion in the literature about designing electronic basis sets for the hydrogen atom for use in multicomponent methods,^50–52^ a consensus on which electronic basis set to use has yet to emerge. Therefore, in this study, we used the conventional correlation-consistent family of electronic basis sets for simplicity.

As mentioned previously, in multicomponent methods, the quantum nuclei no longer have a well-defined position. Therefore, there is not a unique definition of bond distances and angles involving the quantum nuclei. Here, we use two different definitions for the bond distances. The first definition employs the differences between the expectation values of the positions of the quantum nuclei, while the second definition employs the differences between the positions of the nuclear basis function centers. The position expectation values are evaluated with the relaxed multicomponent CCSD protonic density obtained from the Λ and Z-vector equations. This is the same density used for the dipole moments in Section 4.2. Our preferred definition is the first which is what is presented in the body of the study, but we present the second definition in the Supporting Information for completeness.

We calculated all multicomponent CCSD dipoles at the minima on the multicomponent PESs found during the geometry optimizations. We also computed multicomponent rotational constants at the minima. For the rotational constants, we assume that all quantum nuclear mass is located at its position expectation value, which is computed from the relaxed protonic density.

For comparison, we performed single-component CCSD geometry optimizations and dipole moment calculations using identical electronic basis sets with CFOUR.^53^ We also computed vibrationally averaged geometries and rotational constants using second-order vibrational perturbation theory (VPT2),^54^ as implemented in CFOUR. Due to limited computational resources, we were not able to perform VPT2 calculations with all species using the aug-cc-pVQZ basis set. Additionally, CFOUR provides only vibrationally averaged bond distances and not angles.

All geometry optimizations used the geomeTRIC optimizer through its PySCF interface, in translation-rotation-internal coordinates. The optimizer is a quasi-Newton method which makes use of the BFGS algorithm to update an approximate Hessian inside of a trust region. No analytic or numerical second derivatives are computed. We used the default convergence criteria of 10^–6^ Hartree on the energy change, 3 × 10^–4^ and 4.5 × 10^–4^ a.u. on the root-mean-square and maximum gradient components, and 1.2 × 10^–3^ and 1.8 × 10^–3^ a.u. on the root-mean-square and maximum displacements. The coordinates of the protonic basis-function centers are optimized on the same footing as the classical nuclear coordinates.

## 4 Results and Discussion

### 4.1 Geometries

The optimized single-component CCSD, multicomponent CCSD, and VPT2-CCSD geometries for the triatomic systems; HCCH, HNNH, HOOH, and their associated isotopologues; and (HF)_2_ and its associated isotopologues are shown in Tables 1, 2, and 3, respectively.

**Table 1:** Optimized Single-Component CCSD, Multicomponent (MC) CCSD, and VPT2-CCSD Bond Distances (Angstroms) for the Triatomic Systems for the aug-cc-pVDZ (DZ), aug-cc-pVTZ (TZ), and aug-cc-pVQZ (QZ) Electronic Basis Sets

		CCSD			MC CCSD			VPT2-CCSD	
		DZ	TZ	QZ	DZ	TZ	QZ	DZ	TZ
XCN	H–C	1.079	1.058	1.063	1.092	1.071	1.075	1.097	1.077
	D–C	1.079	1.058	1.063	1.088	1.068	1.072	1.092	1.072
XNC	H–N	1.004	0.992	0.992	1.019	1.006	1.003	1.021	1.009
	D–N	1.004	0.992	0.992	1.015	1.003	1.001	1.017	1.005
FXF^–^	H–F	1.148	1.134	1.133	1.162	1.146	1.146	1.178	1.161
	D–F	1.148	1.134	1.133	1.158	1.143	1.143	1.170	1.154

For all systems, multicomponent CCSD and VPT2-CCSD predict longer X-H bond distances than single-component CCSD, showing that both methods can capture the expected effects of vibrational averaging on molecular geometries. In general, VPT2-CCSD predicts slightly longer X-H bond distances than multicomponent CCSD.

For X-D bonds, single-component CCSD predicts the same bond distances as for the analogous X-H bonds for all molecules because the Born–Oppenheimer potential energy surface does not depend on the nuclear mass. Multicomponent CCSD and VPT2-CCSD predict shorter X-D bond distances than the analogous X-H bond distances for all molecules with the bonds shortening by 0.002–0.006 Å. For the deuterated bonds, VPT2-CCSD again predicts longer bond distances than multicomponent CCSD, but the agreement between the two methods is closer with the maximum disagreement being 0.004 Å for the O–D bond distance in HOOD.

We emphasize that VPT2-CCSD and multicomponent CCSD make qualitatively similar predictions about the effects of vibrational averaging but that the multicomponent CCSD values are obtained from a single geometry optimization on the multicomponent potential energy surface, while VPT2-CCSD requires the calculation of fourth-order force constants, which must be done numerically in most cases. It is also possible that there would be better agreement between multicomponent CCSD and VPT2-CCSD if additional electron–proton correlations were included at the triples level as this has been shown to be important for accurate proton affinities.^28,31^ The electronic basis set used for the quantum nuclei is another possible source of the differences found between multicomponent CCSD and VPT2-CCSD. The correlation-consistent basis sets used here are contracted for a classical nucleus at a fixed position. When the nucleus is treated quantum mechanically and delocalizes, a contracted electronic basis centered at the nuclear basis-function center cannot follow that delocalization. This clamps the quantum nucleus and biases the optimized bond distances. Decontraction and further augmentation of the electronic basis at the quantum center have been shown to matter for multicomponent calculations, and the effect on optimized geometries is worth a systematic study.^50,51^ Finally, it is possible that another source of disagreement is that multicomponent CCSD in this study treats only the hydrogen or deuterium nuclei of the systems quantum mechanically.

Multicomponent CCSD predicts similar bond and dihedral angles to single-component CCSD with the maximum difference being less than 1.2° and 1.7° for the bond and dihedral angles, respectively. The bond and dihedral angles of the deuterated form are similar to their nondeuterated analogues with the largest difference being 0.4° for the X_b_-F-X acceptor angle of the (XF)_2_ system.

To test the sensitivity of the results in Tables 1–7 to the choice of protonic basis set, the geometry optimizations and the dipole moment and rotational constant calculations were repeated with the smaller PB4-D protonic basis set and with an uncontracted even-tempered 8s8p8d8f protonic basis set (Tables S10–S23 of the Supporting Information). The 8s8p8d8f results are nearly identical to the PB4-F1 results, indicating that the properties reported here are converged with respect to the protonic basis set, while the smaller PB4-D set lengthens the X-H and X-D bond distances by a few thousandths of an angstrom with correspondingly small changes in the dipole moments and rotational constants. No conclusion of this work depends on the choice of the protonic basis set.

**Table 2:** Optimized Single-Component CCSD, Multicomponent (MC) CCSD, and VPT2-CCSD Bond Distances (Angstroms) and Angles (Degrees) for HCCH, HNNH, HOOH, and their Isotopologues for the aug-cc-pVDZ (DZ), aug-cc-pVTZ (TZ), and aug-cc-pVQZ (QZ) Electronic Basis Sets

		CCSD			MC CCSD			VPT2-CCSD	
		DZ	TZ	QZ	DZ	TZ	QZ	DZ	TZ
XCCX	H–C	1.076	1.056	1.059	1.089	1.069	1.072	1.093	1.074
	D–C	1.076	1.056	1.059	1.085	1.066	1.069	1.089	1.070
XCCY	H–C	1.076	1.056	1.059	1.089	1.069	1.071	1.093	1.074
	D–C	1.076	1.056	1.059	1.086	1.067	1.069	1.089	1.070
XNNX	H–N	1.038	1.024	1.024	1.057	1.041	1.041	1.060	1.046
	θ(H–N–N)	106.1	106.7	106.8	106.3	106.9	107.1		
	D–N	1.038	1.024	1.024	1.051	1.036	1.037	1.054	1.040
	θ(D–N–N)	106.1	106.7	106.8	106.3	106.9	107.0		
XNNY	H–N	1.038	1.024	1.024	1.057	1.041	1.041	1.060	1.046
	D–N	1.038	1.024	1.024	1.051	1.036	1.037	1.054	1.040
	θ(H–N–N)	106.1	106.7	106.8	106.3	106.9	107.1		
	θ(D–N–N)	106.1	106.7	106.8	106.3	106.9	107.0		
XOOX	H–O	0.968	0.960	0.959	0.987	0.973	0.971	0.987	0.979
	θ(H–O–O)	100.1	100.7	100.9	100.1	100.8	101.1		
	O–O	1.460	1.436	1.431	1.464	1.439	1.433	1.473	1.448
	τ(H–O–O–H)	111.9	111.7	111.7	113.2	113.3	112.6		
	D–O	0.968	0.960	0.959	0.981	0.970	0.968	0.982	0.974
	θ(D–O–O)	100.1	100.7	100.9	100.1	100.7	101.0		
	O–O	1.460	1.436	1.431	1.463	1.438	1.433	1.472	1.447
	τ(D–O–O–D)	111.9	111.7	111.7	112.9	113.1	112.5		
XOOY	H–O	0.968	0.960	0.959	0.987	0.973	0.971	0.987	0.979
	D–O	0.968	0.960	0.959	0.982	0.970	0.968	0.982	0.974
	θ(H–O–O)	100.1	100.7	100.9	100.1	100.8	101.1		
	θ(D–O–O)	100.1	100.7	100.9	100.0	100.7	101.0		
	O–O	1.460	1.436	1.431	1.463	1.439	1.433	1.472	1.448
	τ(H–O–O–D)	111.9	111.7	111.7	113.0	113.4	112.7		

**Table 3:** Optimized Single-Component CCSD, Multicomponent (MC) CCSD, and VPT2-CCSD Bond Distances (Angstroms) and Angles (Degrees) for (HF)_2_ and their Isotopologues for the aug-cc-pVDZ (DZ), aug-cc-pVTZ (TZ), and aug-cc-pVQZ (QZ) Electronic Basis Sets^a^

		CCSD			MC CCSD			VPT2-CCSD	
		DZ	TZ	QZ	DZ	TZ	QZ	DZ	TZ
(XF)2	H–F (donor)	0.927	0.921	0.919	0.948	0.932	0.931	0.943	0.936
	H–F (acceptor)	0.925	0.919	0.917	0.944	0.930	0.927	0.941	0.934
	θ(F–H_b–F) bridge	170.0	171.2	170.2	169.4	171.2	170.0		
	θ(H_b–F–H) acceptor	114.5	116.1	116.0	114.2	116.8	116.9		
	D–F (donor)	0.927	0.921	0.919	0.942	0.930	0.928	0.938	0.932
	D–F (acceptor)	0.925	0.919	0.917	0.939	0.928	0.925	0.936	0.930
	θ(F–D_b–F) bridge	170.0	171.2	170.2	169.6	171.3	170.1		
	θ(D_b–F–D) acceptor	114.5	116.1	116.0	114.4	116.7	116.5		
(HF)(DF)	H–F (donor: HF)	0.927	0.921	0.919	0.948	0.933	0.931	0.943	0.936
	D–F (acceptor: DF)	0.925	0.919	0.917	0.939	0.928	0.925	0.936	0.930
	θ(F–H_b–F) bridge	170.0	171.2	170.2	169.5	170.9	169.8		
	θ(H_b–F–D) acceptor	114.5	116.1	116.0	114.2	116.3	116.2		
(DF)(HF)	H–F (acceptor: HF)	0.925	0.919	0.917	0.944	0.930	0.927	0.941	0.934
	D–F (donor: DF)	0.927	0.921	0.919	0.942	0.930	0.928	0.938	0.932
	θ(D_b–F–H) acceptor	114.5	116.1	116.0	114.4	117.2	116.8		
	θ(F–D_b–F) bridge	170.0	171.2	170.2	169.5	171.4	170.2		

a The label “b” indicates the bridging hydrogen or deuterium.

**Table 4:** Dipole Moments (Debye) for the Single-Component CCSD, Multicomponent (MC) CCSD, and VPT2-CCSD Methods for the aug-cc-pVDZ (DZ), aug-cc-pVTZ (TZ), and aug-cc-pVQZ (QZ) Electronic Basis Sets^a^

		CCSD	MC CCSD	VPT2-CCSD	Exp.
	DZ	3.043	3.113	3.014	
HCN	TZ	3.044	3.098	3.016	2.985
	QZ	3.059	3.102		
	DZ	3.043	3.093	3.021	
DCN	TZ	3.044	3.084	3.023	2.991
	QZ	3.059	3.093		
	DZ	3.075	3.158	2.968	
HNC	TZ	3.102	3.169	3.009	3.050
	QZ	3.104	3.156		
	DZ	3.075	3.134	2.995	
DNC	TZ	3.102	3.153	3.032	N/A
	QZ	3.104	3.145		
	DZ	0.000	0.016	0.013	
HCCD	TZ	0.000	0.010	0.009	0.010
	QZ	0.000	0.006		
	DZ	0.000	0.012	0.011	
HNND	TZ	0.000	0.008	0.010	N/A
	QZ	0.000	0.004		
	DZ	1.779	1.818	1.678	
HOOH	TZ	1.774	1.790	1.674	1.573
	QZ	1.787	1.801		
	DZ	1.779	1.813	1.688	
DOOH	TZ	1.774	1.783	1.684	N/A
	QZ	1.787	1.796		
	DZ	1.779	1.807	1.704	
DOOD	TZ	1.774	1.783	1.699	N/A
	QZ	1.787	1.796		
	DZ	3.307	3.513	3.271	
(HF)_2_	TZ	3.365	3.541	3.506	2.989
	QZ	3.344	3.496		
	DZ	3.307	3.458	3.279	
(DF)_2_	TZ	3.365	3.501	3.465	2.992
	QZ	3.344	3.458		
	DZ	3.307	3.489	3.303	
(HF)(DF)	TZ	3.365	3.507	3.509	3.028
	QZ	3.344	3.466		
	DZ	3.307	3.483	3.256	
(DF)(HF)	TZ	3.365	3.530	3.467	3.028
	QZ	3.344	3.479		

a Experimental values are taken from refs 57–61.

**Table 5:** Rotational Constants (MHz) for the Single-Component CCSD, Multicomponent (MC) CCSD, and VPT2-CCSD Methods for the Linear Systems using the aug-cc-pVDZ (DZ), aug-cc-pVTZ (TZ), and aug-cc-pVQZ (QZ) Electronic Basis Sets^a^

			CCSD	MC CCSD	VPT2-CCSD	Exp.
		DZ	43395	43212	43209	
HCN	B	TZ	44922	44730	44734	44316
		QZ	44983	44803		
		DZ	35420	35255	35301	
DCN	B	TZ	36679	36504	36559	36207
		QZ	36694	36524		
		DZ	44367	44149	44230	
HNC	B	TZ	45816	45613	45666	45332
		QZ	45972	45800		
		DZ	37292	37100	37258	
DNC	B	TZ	38477	38287	38427	38153
		QZ	38601	38439		
		DZ	34350	34104	34244	
HCCH	B	TZ	35679	35416	35501	35275
		QZ	35730	35485		
		DZ	28926	28721	28874	
HCCD	B	TZ	30043	29817	29922	29725
		QZ	30069	29859		
		DZ	24720	24550	24701	
DCCD	B	TZ	25674	25483	25591	25419
		QZ	25684	25506		

a Experimental values are taken from refs 62–66.

**Table 6:** Rotational Constants (MHz) for the Single-Component CCSD, Multicomponent (MC) CCSD, and VPT2-CCSD Methods for HNNH, HNND, and DNND using the aug-cc-pVDZ (DZ), aug-cc-pVTZ (TZ), and aug-cc-pVQZ (QZ) Electronic Basis Sets^a^

			CCSD	MC CCSD	VPT2-CCSD	Exp.
		DZ	297952	288979	294115	
	A	TZ	308695	300883	305163	299829
		QZ	309052	301474		
		DZ	38786	38625	38560	
HNNH	B	TZ	39831	39689	39608	39103
		QZ	39887	39757		
		DZ	34319	34071	33973	
	C	TZ	35279	35064	34935	34475
		QZ	35328	35125		
		DZ	218998	214038	216944	
	A	TZ	227185	222768	225339	221373
		QZ	227535	223223		
		DZ	35582	35400	35367	
HNND	B	TZ	36494	36325	36281	35854
		QZ	36531	36363		
		DZ	30609	30376	30299	
	C	TZ	31443	31232	31134	30746
		QZ	31478	31269		
		DZ	178276	175064	176909	
	A	TZ	185145	182238	183929	180613
		QZ	185497	182672		
		DZ	32436	32242	32229	
DNND	B	TZ	33231	33043	33024	32665
		QZ	33253	33066		
		DZ	27443	27227	27167	
	C	TZ	28174	27971	27898	27568
		QZ	28198	27998		

a Experimental values are taken from ref 67.

**Table 7:** Rotational Constants (MHz) for the Single-Component CCSD, Multicomponent (MC) CCSD, and VPT2-CCSD Methods for HOOH, HOOD, and DOOD using the aug-cc-pVDZ (DZ), aug-cc-pVTZ (TZ), and aug-cc-pVQZ (QZ) Electronic Basis Sets^a^

			CCSD	MC CCSD	VPT2-CCSD	Exp.
		DZ	300508	289761	297836	
	A	TZ	307416	300061	305036	301873
		QZ	309021	301936		
		DZ	26141	25981	25842	
HOOH	B	TZ	26932	26812	26660	26192
		QZ	27115	26985		
		DZ	25353	25129	24943	
	C	TZ	26124	25933	25735	25118
		QZ	26302	26127		
		DZ	211483	205383	210025	
	A	TZ	216537	212289	215257	212751
		QZ	217733	213578		
		DZ	24786	24642	24542	
DOOH	B	TZ	25506	25399	25286	24831
		QZ	25669	25545		
		DZ	23547	23355	23194	
	C	TZ	24235	24067	23899	23423
		QZ	24391	24229		
		DZ	163804	159759	162912	
	A	TZ	167817	164989	167060	165085
		QZ	168778	165963		
		DZ	23214	23093	23018	
DOOD	B	TZ	23857	23774	23682	23329
		QZ	23999	23892		
		DZ	22149	21967	21838	
	C	TZ	22776	22614	22479	22019
		QZ	22916	22762		

a Experimental values are taken from refs 68 and 69.

### 4.2 Dipole Moments

High-quality experimental dipole moments exist for many of the test systems in this study, allowing for the comparison of multicomponent CCSD directly to experiment. While there have been benchmarking studies of dipole moments that compared CCSD and DFT dipole moments to reference CCSD­(T) results, we are unaware of any similar large-scale study that has compared directly to experimental dipole moments as this previous benchmarking study notes that its calculated dipole moments lack vibrational corrections.^55^ We note that the accuracy of VPT2 dipole moments compared to experiment has not been thoroughly studied. It has been shown in a recent study that VPT2-DFT dipole moments are not substantially more accurate than standard DFT dipole moments,^56^ but as shown below, the performance of VPT2-CCSD is more accurate than single-component CCSD for the small number of test systems in this study.

The CCSD, multicomponent CCSD, VPT2-CCSD, and, when available, experimental dipole moments are shown in Table 4. We highlight a novel aspect of multicomponent CCSD and VPT2-CCSD compared to single-component CCSD in that they predict small dipole moments for HCCD and HNND. As HCCD and HNND have charge-inversion symmetry at the single-component CCSD level of theory, single-component CCSD predicts both molecules to have no dipole moment.

In general, for systems in which single-component CCSD predicts a nonzero dipole moment, multicomponent CCSD predicts larger dipole moments than single-component CCSD, while VPT2-CCSD predicts smaller dipole moments. The source of this difference is unknown at this time, but similar to the differences in geometries, this difference is likely in part due to an incomplete treatment of electron–proton correlation and could be improved by including higher-order corrections to the electron–proton correlation energy.

For most of the dipole values presented here, single-component CCSD overestimates the dipole such that multicomponent CCSD performs worse than single-component CCSD, while VPT2-CCSD performs better than both other methods. Even though multicomponent CCSD performs more poorly than single-component CCSD and VPT2-CCSD for the prediction of dipole moments, it still performs adequately, with a maximum absolute error of less than 0.3 D for the covalently bonded systems, however, given that it is more computationally expensive than single-component CCSD, we do not recommend its use for computing dipole moments based on the results in this study.

### 4.3 Rotational Constants

The single-component CCSD, multicomponent CCSD, VPT2-CCSD, and experimental rotational constants for the linear systems, HNNH and related isotopologues, and HOOH and related isotopologues are presented in Tables 5, 6, and 7, respectively. In general, multicomponent CCSD and VPT2-CCSD produce similar rotational constants with the maximum difference between them being 2.7% and 1.6% with the aug-cc-pVDZ and aug-cc-pVTZ electronic basis sets, respectively.

For the linear systems, multicomponent CCSD and VPT2-CCSD predict rotational constants that are smaller than those of single-component CCSD, which is consistent with their longer predicted bond lengths. For the aug-cc-pVTZ and aug-cc-pVQZ electronic basis sets, single-component CCSD predicts rotational constants that are larger than the experiment, and multicomponent CCSD performs better than single-component CCSD for every linear system, as does VPT2-CCSD with the aug-cc-pVTZ basis set. With the aug-cc-pVDZ electronic basis set, the single-component CCSD rotational constants already lie below the experiment, and vibrational averaging moves them further from the experiment.

For the nonlinear systems, multicomponent CCSD performs better than single-component CCSD for all 18 rotational constants with the aug-cc-pVTZ and aug-cc-pVQZ electronic basis sets. The largest improvements are in the A constants, which are determined almost entirely by the positions of the hydrogen nuclei. With the aug-cc-pVTZ electronic basis set, multicomponent CCSD is closer to experiment than VPT2-CCSD for all six A constants, whereas VPT2-CCSD is closer for the B and C constants. Over the 25 constants presented in Tables 5–7 with experimental values, the mean absolute errors with the aug-cc-pVTZ electronic basis set are 1708 MHz for single-component CCSD, 1101 MHz for VPT2-CCSD, and 579 MHz for multicomponent CCSD. With the aug-cc-pVQZ electronic basis set, the mean absolute errors are 1966 MHz for single-component CCSD and 684 MHz for multicomponent CCSD. With the aug-cc-pVDZ electronic basis set, multicomponent CCSD is further from the experiment than single-component CCSD. With that basis set, the bond distances are already too long and the rotational constants lie below experiment, so vibrational averaging increases the error, whereas with the larger basis sets, the constants lie above experiment and vibrational averaging reduces it.

More benchmarking in the future is needed before a definitive conclusion is reached, but based on the results presented in this study, multicomponent CCSD appears to be a useful method for the calculation of vibrationally averaged rotational constants, particularly for calculations with larger electronic basis sets, given that it has the same formal scaling as single-component CCSD and can compute these rotational constants from a single geometry optimization on the multicomponent potential energy surface.

### 4.4 Protonic Densities

Finally, we compare the multicomponent CCSD protonic densities of HCN and FHF^–^ to exact grid-based results. Three multicomponent densities are considered. The multicomponent HF density, the unrelaxed density built from the T and Λ amplitudes, and the relaxed density, which adds the orbital response from the Z-vector equations and is the density contracted for the analytic gradient and the dipole moments. The exact reference is the proton density on the clamped-nuclei single-component CCSD surface, obtained by scanning the CCSD energy over proton positions and solving the protonic Schrödinger equation on a three-dimensional Fourier grid with the nuclear proton mass. All calculations use the aug-cc-pVTZ electronic and PB4-F1 protonic basis sets at the optimized multicomponent CCSD geometries.

Table 8 reports the root-mean-square deviation of each multicomponent density from the exact density, evaluated pointwise over the Fourier grid. Correlation reduces the deviation by a factor of 2.2–2.9, as the multicomponent HF density is too compact, with a peak height several times that of the exact one. The relaxed and unrelaxed densities are equally close to the exact density, differing by at most 0.013 Bohr^–3^. Orbital relaxation instead shifts the center of the density, by 2.8 mÅ for HCN, while changing the RMS width by less than 1 mÅ. For FHF^–^, the center is fixed by symmetry. Orbital relaxation therefore changes the position of the protonic density rather than its shape, and this position shift is what produces the longer vibrationally averaged bond distances in Tables 1–3 and the improved rotational constants in Tables 5–7.

**Table 8:** Root-Mean-Square Deviation (Bohr^–3^) of the Multicomponent Protonic Densities of HCN and FHF^–^ from the Exact Fourier Grid Density, Evaluated Pointwise over the Fourier Grid, with the aug-cc-pVTZ Electronic and PB4-F1 Protonic Basis Sets at the Optimized Multicomponent CCSD Geometries

	HF	Unrelaxed CCSD	Relaxed CCSD
HCN	0.572	0.259	0.264
FHF^–^	0.582	0.204	0.217

## 5 Conclusions

An analytic nuclear energy gradient is derived and implemented for multicomponent CCSD. Using the analytic gradient, optimized geometries, dipole moments, and rotational constants were calculated, and it was shown that multicomponent CCSD correctly includes the physics needed to describe vibrationally averaged geometries and rotational constants. The relaxed protonic density matrices obtained from the Z-vector equations improve the multicomponent CCSD vibrationally averaged geometries and rotational constants relative to those obtained from the multicomponent HF protonic density and give a more rigorous route to the proton densities by which multicomponent wave function methods are judged. With an analytic first derivative available, a seminumerical multicomponent CCSD Hessian becomes practical, which would make harmonic frequencies, infrared intensities, and transition-state searches accessible for systems in which nuclear quantum effects are important. Combined with the overall importance of analytic nuclear energy gradients for geometry optimization, this makes it likely that the multicomponent CCSD analytic nuclear energy gradient will enable diverse applications of multicomponent coupled cluster in the future.

## Supplementary Material



## Data Availability

The data underlying this study are available in the published article and its Supporting Information.

## Notes

Comparison of the analytic nuclear energy gradient and dipole moments against finite differences, results from Section 4.1 using the protonic basis function centers as the location of the quantum protons, and additional calculations with the PB4-D and 8s8p8d8f protonic basis sets are available (PDF)
