# Supplementary material for: An Analytic Nuclear Energy Gradient for Multicomponent CCSD
Source: J Chem Theory Comput. 2026 Aug 23;22(17):8813–24. doi: 10.1021/acs.jctc.6c01090 (PMC13564356; doi:10.1021/acs.jctc.6c01090)
Supplement: Supplementary file 1 [file ct6c01090_si_001.pdf]

Supplemental Material for "An Analytic Nuclear Energy Gradient for Multicomponent CCSD"

Gabrielle B. Tucker[1] and Kurt R. Brorsen[1]

Correspondence to Kurt R. Brorsen (Email: brorsenk@missouri.edu)

[1]Department of Chemistry, University of Missouri, Columbia, Missouri 65203, United States of America

atom #	symbol	role	x	y	z
0	H	quantum	0.000000	0.000000	0.000000
1	C	classical	0.000000	0.000000	1.150000
2	N	classical	0.000000	0.000000	2.450000

Table S1: HCN geometry for numerical gradients and dipole validation. All coordinates in angstroms.

atom #	symbol	role	x	y	z
0	F	classical	0.000000	0.000000	0.000000
1	H	quantum	0.000000	0.000000	0.950000
2	F	classical	0.000000	0.000000	2.800000
3	H	quantum	0.892708	0.000000	3.124919

Table S2: $(HF)_2$ geometry for numerical gradients and dipole validation. All coordinates in angstroms.

atom #	symbol	role	x	y	z
0	H	quantum	0.000000	0.860000	0.000000
1	F	classical	0.000000	1.788000	0.000000
2	H	quantum	0.745000	-0.430000	0.000000
3	F	classical	1.549000	-0.894000	0.000000
4	H	quantum	-0.745000	-0.430000	0.000000
5	F	classical	-1.549000	-0.894000	0.000000

Table S3: $(HF)_3$ geometry for numerical gradients validation. All coordinates in angstroms.

aug-cc-pVDZ

atom #	axis	analytic	FD h = 1e-02	FD h = 1e-03	FD h = 1e-04
0	x	8.8309986336E-17	0.0000000000E+00	0.0000000000E+00	0.0000000000E+00
0	y	3.4256804817E-17	0.0000000000E+00	0.0000000000E+00	0.0000000000E+00
0	z	-2.4421237901E-02	-2.4417985245E-02	-2.4421205417E-02	-2.4421239075E-02
1	x	-1.4037678097E-16	0.0000000000E+00	0.0000000000E+00	0.0000000000E+00
1	y	-3.8512942793E-17	0.0000000000E+00	0.0000000000E+00	0.0000000000E+00
1	z	-1.7368982707E-01	-1.7365885341E-01	-1.7368951718E-01	-1.7368982377E-01
2	x	5.2066794632E-17	0.0000000000E+00	0.0000000000E+00	0.0000000000E+00
2	y	4.2561379766E-18	0.0000000000E+00	0.0000000000E+00	0.0000000000E+00
2	z	1.9811106497E-01	1.9807680499E-01	1.9811072249E-01	1.9811106490E-01
max | analytic - FD |			3.43E-05	3.42E-07	3.30E-09

aug-cc-pVTZ

atom #	axis	analytic	FD h = 1e-02	FD h = 1e-03	FD h = 1e-04
0	x	5.7181016421E-16	0.0000000000E+00	0.0000000000E+00	0.0000000000E+00
0	y	-5.2567391533E-15	0.0000000000E+00	0.0000000000E+00	0.0000000000E+00
0	z	-1.9811322226E-02	-1.9806279085E-02	-1.9811271528E-02	-1.9811319873E-02
1	x	-6.1444088467E-16	0.0000000000E+00	0.0000000000E+00	0.0000000000E+00
1	y	7.9513229974E-15	0.0000000000E+00	0.0000000000E+00	0.0000000000E+00
1	z	-2.0548598360E-01	-2.0545953057E-01	-2.0548571934E-01	-2.0548597902E-01
2	x	4.2630720462E-17	0.0000000000E+00	0.0000000000E+00	0.0000000000E+00
2	y	-2.6945838441E-15	0.0000000000E+00	0.0000000000E+00	0.0000000000E+00
2	z	2.2529730583E-01	2.2526522343E-01	2.2529698430E-01	2.2529730231E-01
max | analytic - FD |			3.21E-05	3.22E-07	4.58E-09

Table S4: HCN gradient values (a.u.) for the analytic gradient and numerical gradient (FD) with the aug-cc-pVDZ and aug-cc-PVTZ electronic and PB4-F1 protonic basis sets.

aug-cc-pVDZ

axis	analytic	FD h_F = 1e-03	FD h_F = 1e-04	FD h_F = 1e-05
x	-2.2310345125E-16	0.0000000000E+00	0.0000000000E+00	0.0000000000E+00
y	8.8140519167E-16	0.0000000000E+00	0.0000000000E+00	0.0000000000E+00
z	-1.2243482294E+00	-1.2243474869E+00	-1.2243482232E+00	-1.2243482267E+00
max | analytic - FD |		7.43E-07	6.28E-09	2.73E-09

aug-cc-pVTZ

axis	analytic	FD h_F = 1e-03	FD h_F = 1e-04	FD h_F = 1e-05
x	6.2836794353E-15	0.0000000000E+00	0.0000000000E+00	0.0000000000E+00
y	-7.5927934324E-14	0.0000000000E+00	0.0000000000E+00	0.0000000000E+00
z	-1.2184282357E+00	-1.2184286752E+00	-1.2184282398E+00	-1.2184282454E+00
max | analytic - FD |		4.39E-07	4.05E-09	9.66E-09

Table S5: HCN dipole values (a.u.) for the analytic dipole and numerical dipole (FD) with the aug-cc-pVDZ and aug-cc-PVTZ electronic and PB4-F1 protonic basis sets.

aug-cc-pVDZ

atom #	axis	analytic	FD h = 1e-02	FD h = 1e-03	FD h = 1e-04
0	x	-1.2003197159E-03	-1.2002952417E-03	-1.2003196304E-03	-1.2003235383E-03
0	y	-5.4440508043E-17	0.0000000000E+00	0.0000000000E+00	0.0000000000E+00
0	z	-2.1451695647E-02	-2.1444654477E-02	-2.1451625116E-02	-2.1451694465E-02
1	x	2.2838650016E-03	2.2838379238E-03	2.2838646743E-03	2.2838680991E-03
1	y	6.0910032697E-17	0.0000000000E+00	0.0000000000E+00	0.0000000000E+00
1	z	2.0095266123E-02	2.0087972966E-02	2.0095192909E-02	2.0095266109E-02
2	x	-2.2774793390E-02	-2.2769498584E-02	-2.2774740344E-02	-2.2774790267E-02
2	y	-1.5421017366E-17	0.0000000000E+00	0.0000000000E+00	0.0000000000E+00
2	z	-5.5704083593E-03	-5.5721473331E-03	-5.5704257420E-03	-5.5704100532E-03
3	x	2.1691248104E-02	2.1685901146E-02	2.1691194647E-02	2.1691250254E-02
3	y	8.9514927115E-18	0.0000000000E+00	0.0000000000E+00	0.0000000000E+00
3	z	6.9268378837E-03	6.9279972678E-03	6.9268494798E-03	6.9268327252E-03
max | analytic - FD |			7.29E-06	7.32E-08	5.16E-09

aug-cc-pVTZ

atom #	axis	analytic	FD h = 1e-02	FD h = 1e-03	FD h = 1e-04
0	x	-1.1512071377E-03	-1.1511692989E-03	-1.1512064191E-03	-1.1512061349E-03
0	y	-6.4675149928E-17	0.0000000000E+00	0.0000000000E+00	0.0000000000E+00
0	z	-1.3402886817E-02	-1.3401741246E-02	-1.3402875439E-02	-1.3402888328E-02
1	x	2.2502921796E-03	2.2502152532E-03	2.2502913311E-03	2.2502894126E-03
1	y	7.2930372700E-17	0.0000000000E+00	0.0000000000E+00	0.0000000000E+00
1	z	1.1652879563E-02	1.1651787399E-02	1.1652868778E-02	1.1652883245E-02
2	x	-1.3645378660E-02	-1.3644602359E-02	-1.3645370970E-02	-1.3645381216E-02
2	y	7.5115429344E-17	0.0000000000E+00	0.0000000000E+00	0.0000000000E+00
2	z	-1.7638725697E-03	-1.7649123663E-03	-1.7638831196E-03	-1.7638726035E-03
3	x	1.2546293618E-02	1.2545457483E-02	1.2546285404E-02	1.2546289270E-02
3	y	-8.3370652117E-17	0.0000000000E+00	0.0000000000E+00	0.0000000000E+00
3	z	3.5138798235E-03	3.5144821283E-03	3.5138859573E-03	3.5138806709E-03
max | analytic - FD |			1.15E-06	1.14E-08	4.35E-09

Table S6: $(HF)_2$ gradient values (a.u.) for the analytic gradient and numerical gradient (FD) with the aug-cc-pVDZ and aug-cc-PVTZ electronic and PB4-F1 protonic basis sets.

aug-cc-pVDZ

axis	analytic	FD h_F = 1e-03	FD h_F = 1e-04	FD h_F = 1e-05
x	7.3160677619E-01	7.3160666960E-01	7.3160677516E-01	7.3160679648E-01
y	2.0297511450E-16	0.0000000000E+00	0.0000000000E+00	0.0000000000E+00
z	1.2455750950E+00	1.2455809418E+00	1.2455751553E+00	1.2455751261E+00
max | analytic - FD |		5.85E-06	6.04E-08	3.11E-08

aug-cc-pVTZ

axis	analytic	FD h_F = 1e-03	FD h_F = 1e-04	FD h_F = 1e-05
x	7.0765240177E-01	7.0765245096E-01	7.0765240608E-01	7.0765236444E-01
y	-2.7989345197E-15	0.0000000000E+00	0.0000000000E+00	0.0000000000E+00
z	1.2086203746E+00	1.2086263890E+00	1.2086204404E+00	1.2086203967E+00
max | analytic - FD |		6.01E-06	6.58E-08	3.73E-08

Table S7: $(HF)_2$ dipole values (a.u.) for the analytic dipole and numerical dipole (FD) with the aug-cc-pVDZ and aug-cc-PVTZ electronic and PB4-F1 protonic basis sets.

cc-pVDZ

atom #	axis	analytic	FD h = 2e-03
0	x	3.335327E-15	-1.989520E-10
0	y	-3.202376E-02	-3.202376E-02
0	z	1.490564E-17	9.473903E-12
1	x	-3.328834E-15	5.684342E-11
1	y	9.246130E-03	9.246130E-03
1	z	-4.407489E-18	-4.736952E-12
2	x	-2.784637E-02	-2.784637E-02
2	y	1.608214E-02	1.608214E-02
2	z	9.617190E-18	7.105427E-12
3	x	8.138442E-03	8.138441E-03
3	y	-4.693320E-03	-4.693320E-03
3	z	5.018589E-18	2.368476E-12
4	x	2.784637E-02	2.784637E-02
4	y	1.608214E-02	1.608214E-02
4	z	3.921510E-18	4.736952E-12
5	x	-8.138442E-03	-8.138442E-03
5	y	-4.693320E-03	-4.693321E-03
5	z	-2.905544E-17	-9.473903E-12

Table S8: Analytic multicomponent CCSD nuclear gradient (a.u.) of cyclic $(HF)_3$ with three quantum protons compared with five-point finite differences ($h = 2\times10^{-3}$ bohr), cc-pVDZ electronic and PB4-F1 protonic basis sets.

	MC CCSD							
		DZ	TZ	QZ		DZ	TZ	QZ
XCN	H-C	1.056	1.017	1.042	D-C	1.059	1.033	1.053
XNC	H-N	0.981	0.960	0.959	D-N	0.983	0.972	0.975
FXF^-	H-F	1.162	1.146	1.146	D-F	1.158	1.143	1.143
XCCX	H-C	1.054	1.021	1.039	D-C	1.057	1.035	1.049
XCCY	H-C	1.053	1.021	1.035	D-C	1.058	1.036	1.050
XNNX	H-N	1.027	1.001	1.010	D-N	1.027	1.009	1.018
	θ(H-N-N)	106.2	106.8	107.0	θ(D-N-N)	106.2	106.8	106.9
XNNY	H-N	1.027	1.001	1.007	D-N	1.027	1.009	1.019
	θ(H-N-N)	106.2	106.8	106.9	θ(D-N-N)	106.2	106.8	106.9
XOOX	H-O	0.950	0.925	0.933	D-O	0.951	0.938	0.947
	θ(H-O-O)	100.0	100.6	101.0	θ(D-O-O)	100.0	100.6	100.9
	τ(H-O-O-H)	113.1	113.3	112.6	τ(D-O-O-D)	112.9	113.1	112.4
XOOY	H-O	0.950	0.925	0.931	D-O	0.952	0.938	0.948
	θ(H-O-O)	100.0	100.6	100.9	θ(D-O-O)	100.0	100.6	100.9
	τ(H-O-O-D)	113.0	113.3	112.6				
(XF)2	H-F (donor)	0.898	0.875	0.888	D-F (donor)	0.900	0.895	0.905
	H-F (acceptor)	0.893	0.873	0.882	D-F (acceptor)	0.896	0.892	0.901
	θ(F-H_b-F) bridge	169.8	171.7	170.3	θ(F-D_b-F) bridge	169.9	171.6	170.3
	θ(H_b-F-H) acceptor	113.9	116.5	116.6	θ(D_b-F-D) acceptor	114.1	116.5	116.3
(HF)(DF)	H-F (donor: HF)	0.898	0.876	0.887	D-F (acceptor: DF)	0.896	0.893	0.901
	θ(F-H_b-F) bridge	169.8	171.3	170.2	θ(H_b-F-D) acceptor	113.9	116.0	115.9
(DF)(HF)	H-F (acceptor: HF)	0.893	0.875	0.880	D-F (donor: DF)	0.900	0.896	0.905
	θ(D_b-F-H) acceptor	114.1	117.0	116.6	θ(F-D_b-F) bridge	169.8	171.6	170.4

Table S9: Bond distances (angstroms) and angles (degrees) using the coordinates of the protonic basis function center as the position of the quantum nucleus.

		CCSD			MC CCSD			VPT2-CCSD	
		DZ	TZ	QZ	DZ	TZ	QZ	DZ	TZ
XCN	H-C	1.079	1.058	1.063	1.093	1.073	1.078	1.097	1.077
	D-C	1.079	1.058	1.063	1.089	1.069	1.074	1.092	1.072
XNC	H-N	1.004	0.992	0.992	1.020	1.007	1.006	1.021	1.009
	D-N	1.004	0.992	0.992	1.015	1.004	1.003	1.017	1.005
FXF-	H-F	1.148	1.134	1.133	1.163	1.147	1.146	1.178	1.161
	D-F	1.148	1.134	1.133	1.159	1.143	1.142	1.170	1.154

Table S10: Optimized single-component CCSD, multicomponent (MC) CCSD, and VPT2-CCSD bond distances (angstroms) for the triatomic systems, as in Table 1 of the manuscript, with the PB4-D protonic basis set (aug-cc-pVDZ, aug-cc-pVTZ, aug-cc-pVQZ electronic basis sets).

		CCSD			MC CCSD			VPT2-CCSD	
		DZ	TZ	QZ	DZ	TZ	QZ	DZ	TZ
XCCX	H-C	1.076	1.056	1.059	1.090	1.071	1.074	1.093	1.074
	D-C	1.076	1.056	1.059	1.086	1.067	1.070	1.089	1.070
XCCY	H-C	1.076	1.056	1.059	1.090	1.071	1.074	1.093	1.074
	D-C	1.076	1.056	1.059	1.086	1.067	1.070	1.089	1.070
XNNX	H-N	1.038	1.024	1.024	1.057	1.042	1.043	1.060	1.046
	th(H-N-N)	106.1	106.7	106.8	106.3	106.9	107.0		
	D-N	1.038	1.024	1.024	1.051	1.037	1.038	1.054	1.040
	th(D-N-N)	106.1	106.7	106.8	106.3	106.9	107.0		
XNNY	H-N	1.038	1.024	1.024	1.057	1.042	1.043	1.060	1.046
	D-N	1.038	1.024	1.024	1.052	1.037	1.038	1.054	1.040
	th(H-N-N)	106.1	106.7	106.8	106.3	106.9	107.1		
	th(D-N-N)	106.1	106.7	106.8	106.3	106.9	107.0		
XOOX	H-O	0.968	0.960	0.959	0.987	0.974	0.974	0.987	0.979
	th(H-O-O)	100.1	100.7	100.9	100.1	100.8	101.1		
	O-O	1.460	1.436	1.431	1.464	1.439	1.433	1.473	1.448
	tau(H-O-O-H)	111.9	111.7	111.7	113.1	113.4	112.9		
	D-O	0.968	0.960	0.959	0.982	0.971	0.970	0.982	0.974
	th(D-O-O)	100.1	100.7	100.9	100.1	100.7	101.0		
	O-O	1.460	1.436	1.431	1.463	1.438	1.433	1.472	1.447
	tau(D-O-O-D)	111.9	111.7	111.7	112.8	113.0	112.5		
XOOY	H-O	0.968	0.960	0.959	0.987	0.974	0.974	0.987	0.979
	D-O	0.968	0.960	0.959	0.982	0.971	0.970	0.982	0.974
	th(H-O-O)	100.1	100.7	100.9	100.1	100.8	101.1		
	th(D-O-O)	100.1	100.7	100.9	100.0	100.7	101.0		
	O-O	1.460	1.436	1.431	1.463	1.439	1.433	1.472	1.448
	tau(H-O-O-D)	111.9	111.7	111.7	113.0	113.1	112.5		

Table S11: Optimized single-component CCSD, multicomponent (MC) CCSD, and VPT2-CCSD bond distances (angstroms) and angles (degrees) for HCCH, HNNH, HOOH, and their isotopologues, as in Table 2 of the manuscript, with the PB4-D protonic basis set (aug-cc-pVDZ, aug-cc-pVTZ, aug-cc-pVQZ electronic basis sets).

		CCSD			MC CCSD			VPT2-CCSD	
		DZ	TZ	QZ	DZ	TZ	QZ	DZ	TZ
(XF)2	H-F (donor)	0.927	0.921	0.919	0.949	0.935	0.934	0.943	0.936
	H-F (acceptor)	0.925	0.919	0.917	0.945	0.933	0.931	0.941	0.934
	th(F-Hb-F) bridge	170.0	171.2	170.2	169.5	171.4	169.7		
	th(Hb-F-H) acceptor	114.5	116.1	116.0	114.2	116.5	115.8		
	D-F (donor)	0.927	0.921	0.919	0.943	0.932	0.930	0.938	0.932
	D-F (acceptor)	0.925	0.919	0.917	0.939	0.929	0.927	0.936	0.930
	th(F-Db-F) bridge	170.0	171.2	170.2	169.6	171.4	170.3		
	th(Db-F-D) acceptor	114.5	116.1	116.0	114.3	116.6	116.0		
(HF)(DF)	H-F (donor: HF)	0.927	0.921	0.919	0.949	0.935	0.934	0.943	0.936
	D-F (acceptor: DF)	0.925	0.919	0.917	0.940	0.929	0.927	0.936	0.930
	th(F-Hb-F) bridge	170.0	171.2	170.2	169.5	171.4	170.0		
	th(Hb-F-D) acceptor	114.5	116.1	116.0	114.2	116.2	115.9		
(DF)(HF)	H-F (acceptor: HF)	0.925	0.919	0.917	0.945	0.932	0.931	0.941	0.934
	D-F (donor: DF)	0.927	0.921	0.919	0.943	0.932	0.931	0.938	0.932
	th(Db-F-H) acceptor	114.5	116.1	116.0	114.4	116.9	115.9		
	th(F-Db-F) bridge	170.0	171.2	170.2	169.6	171.6	169.8		

Table S12: Optimized single-component CCSD, multicomponent (MC) CCSD, and VPT2-CCSD bond distances (angstroms) and angles (degrees) for (HF)2 and its isotopologues, as in Table 3 of the manuscript, with the PB4-D protonic basis set (aug-cc-pVDZ, aug-cc-pVTZ, aug-cc-pVQZ electronic basis sets).

	MC CCSD		
	DZ	TZ	QZ
HCN	3.114	3.100	3.106
DCN	3.094	3.085	3.095
HNC	3.160	3.173	3.164
DNC	3.135	3.155	3.150
HCCD	0.016	0.011	0.008
HNND	0.011	0.008	0.004
HOOH	1.821	1.790	1.799
DOOH	1.815	1.789	1.802
DOOD	1.808	1.785	1.798
(HF)2	3.516	3.551	3.486
(DF)2	3.459	3.507	3.463
(HF)(DF)	3.491	3.525	3.480
(DF)(HF)	3.485	3.535	3.468

Table S13: Multicomponent CCSD dipole moments (Debye) with the PB4-D protonic basis set, as in Table 4 of the manuscript. The single-component CCSD and experimental values are in Table 4.

			CCSD	MC CCSD	VPT2-CCSD	Exp.
		DZ	43395	43202	43209	
HCN	B	TZ	44922	44710	44734	44316
		QZ	44983	44762		
		DZ	35420	35250	35301	
DCN	B	TZ	36679	36489	36559	36207
		QZ	36694	36496		
		DZ	44367	44143	44230	
HNC	B	TZ	45816	45591	45666	45332
		QZ	45972	45759		
		DZ	37292	37093	37258	
DNC	B	TZ	38477	38264	38427	38153
		QZ	38601	38401		
		DZ	34350	34090	34244	
HCCH	B	TZ	35679	35392	35501	35275
		QZ	35730	35441		
		DZ	28926	28712	28874	
HCCD	B	TZ	30043	29800	29922	29725
		QZ	30069	29824		
		DZ	24720	24542	24701	
DCCD	B	TZ	25674	25468	25591	25419
		QZ	25684	25478		

Table S14: Single-component CCSD, multicomponent (MC) CCSD, and VPT2-CCSD rotational constants (MHz) for the linear systems, as in Table 5 of the manuscript, with the PB4-D protonic basis set.

			CCSD	MC CCSD	VPT2-CCSD	Exp.
		DZ	297952	288573	294115	
HNNH	A	TZ	308695	300112	305163	299829
		QZ	309052	300111		
		DZ	38786	38628	38560	
	B	TZ	39831	39693	39608	39103
		QZ	39887	39751		
		DZ	34319	34068	33973	
	C	TZ	35279	35057	34935	34475
		QZ	35328	35102		
		DZ	218998	213810	216944	
HNND	A	TZ	227185	222450	225339	221373
		QZ	227535	222570		
		DZ	35582	35402	35367	
	B	TZ	36494	36328	36281	35854
		QZ	36531	36367		
		DZ	30609	30373	30299	
	C	TZ	31443	31228	31134	30746
		QZ	31478	31259		
		DZ	178276	174915	176909	
DNND	A	TZ	185145	182024	183929	180613
		QZ	185497	182299		
		DZ	32436	32242	32229	
	B	TZ	33231	33039	33024	32665
		QZ	33253	33060		
		DZ	27443	27224	27167	
	C	TZ	28174	27963	27898	27568
		QZ	28198	27985		

Table S15: Single-component CCSD, multicomponent (MC) CCSD, and VPT2-CCSD rotational constants (MHz) for HNNH, HNND, and DNND, as in Table 6 of the manuscript, with the PB4-D protonic basis set.

			CCSD	MC CCSD	VPT2-CCSD	Exp.
		DZ	300508	289307	297836	
HOOH	A	TZ	307416	299195	305036	301873
		QZ	309021	300095		
		DZ	26141	25979	25842	
	B	TZ	26932	26813	26660	26192
		QZ	27115	26992		
		DZ	25353	25130	24943	
	C	TZ	26124	25931	25735	25118
		QZ	26302	26120		
		DZ	211483	205143	210025	
DOOH	A	TZ	216537	211802	215257	212751
		QZ	217733	212605		
		DZ	24786	24640	24542	
	B	TZ	25506	25390	25286	24831
		QZ	25669	25547		
		DZ	23547	23355	23194	
	C	TZ	24235	24065	23899	23423
		QZ	24391	24232		
		DZ	163804	159602	162912	
DOOD	A	TZ	167817	164609	167060	165085
		QZ	168778	165392		
		DZ	23214	23091	23018	
	B	TZ	23857	23762	23682	23329
		QZ	23999	23886		
		DZ	22149	21967	21838	
	C	TZ	22776	22605	22479	22019
		QZ	22916	22753		

Table S16: Single-component CCSD, multicomponent (MC) CCSD, and VPT2-CCSD rotational constants (MHz) for HOOH, DOOH, and DOOD, as in Table 7 of the manuscript, with the PB4-D protonic basis set.

8s8p8d8f Protonic Basis Set Calculations

The uncontracted even-tempered 8s8p8d8f protonic basis set has exponents from $2\sqrt{2}$ to 32 in successive ratios of $\sqrt{2}$. For the deuterated isotopologues the protonic exponents of each basis set were scaled by the square root of the ratio of the nuclear masses.

		CCSD		MC CCSD		VPT2-CCSD	
		DZ	TZ	DZ	TZ	DZ	TZ
XCN	H-C	1.079	1.058	1.092	1.071	1.097	1.077
	D-C	1.079	1.058	1.088	1.068	1.092	1.072
XNC	H-N	1.004	0.992	1.019	1.005	1.021	1.009
	D-N	1.004	0.992	1.015	1.002	1.017	1.005
FXF-	H-F	1.148	1.134	1.162	1.147	1.178	1.161
	D-F	1.148	1.134	1.158	1.143	1.170	1.154

Table S17: Optimized single-component CCSD, multicomponent (MC) CCSD, and VPT2-CCSD bond distances (angstroms) for the triatomic systems, as in Table 1 of the manuscript, with the 8s8p8d8f protonic basis set (aug-cc-pVDZ, aug-cc-pVTZ electronic basis sets).

		CCSD		MC CCSD		VPT2-CCSD	
		DZ	TZ	DZ	TZ	DZ	TZ
XCCX	H-C	1.076	1.056	1.089	1.069	1.093	1.074
	D-C	1.076	1.056	1.085	1.066	1.089	1.070
XCCY	H-C	1.076	1.056	1.089	1.069	1.093	1.074
	D-C	1.076	1.056	1.085	1.066	1.089	1.070
XNNX	H-N	1.038	1.024	1.057	1.041	1.060	1.046
	th(H-N-N)	106.1	106.7	106.3	107.0		
	D-N	1.038	1.024	1.051	1.036	1.054	1.040
	th(D-N-N)	106.1	106.7	106.3	106.9		
XNNY	H-N	1.038	1.024	1.056	1.041	1.060	1.046
	D-N	1.038	1.024	1.051	1.036	1.054	1.040
	th(H-N-N)	106.1	106.7	106.3	107.0		
	th(D-N-N)	106.1	106.7	106.3	106.9		
XOOX	H-O	0.968	0.960	0.987	0.973	0.987	0.979
	th(H-O-O)	100.1	100.7	100.1	100.8		
	O-O	1.460	1.436	1.464	1.439	1.473	1.448
	tau(H-O-O-H)	111.9	111.7	113.1	113.4		
	D-O	0.968	0.960	0.981	0.969	0.982	0.974
	th(D-O-O)	100.1	100.7	100.1	100.7		
	O-O	1.460	1.436	1.463	1.438	1.472	1.447
	tau(D-O-O-D)	111.9	111.7	112.9	113.1		
XOOY	H-O	0.968	0.960	0.987	0.973	0.987	0.979
	D-O	0.968	0.960	0.981	0.969	0.982	0.974
	th(H-O-O)	100.1	100.7	100.1	100.8		
	th(D-O-O)	100.1	100.7	100.0	100.7		
	O-O	1.460	1.436	1.463	1.439	1.472	1.448
	tau(H-O-O-D)	111.9	111.7	113.0	113.3		

Table S18: Optimized single-component CCSD, multicomponent (MC) CCSD, and VPT2-CCSD bond distances (angstroms) and angles (degrees) for HCCH, HNNH, HOOH, and their isotopologues, as in Table 2 of the manuscript, with the 8s8p8d8f protonic basis set (aug-cc-pVDZ, aug-cc-pVTZ electronic basis sets).

		CCSD		MC CCSD		VPT2-CCSD	
		DZ	TZ	DZ	TZ	DZ	TZ
(XF)2	H-F (donor)	0.927	0.921	0.948	0.932	0.943	0.936
	H-F (acceptor)	0.925	0.919	0.944	0.929	0.941	0.934
	th(F-Hb-F) bridge	170.0	171.2	169.4	171.3		
	th(Hb-F-H) acceptor	114.5	116.1	114.2	116.8		
	D-F (donor)	0.927	0.921	0.942	0.930	0.938	0.932
	D-F (acceptor)	0.925	0.919	0.939	0.928	0.936	0.930
	th(F-Db-F) bridge	170.0	171.2	169.5	171.3		
	th(Db-F-D) acceptor	114.5	116.1	114.4	116.7		
(HF)(DF)	H-F (donor: HF)	0.927	0.921	0.947	0.932	0.943	0.936
	D-F (acceptor: DF)	0.925	0.919	0.939	0.928	0.936	0.930
	th(F-Hb-F) bridge	170.0	171.2	169.4	171.1		
	th(Hb-F-D) acceptor	114.5	116.1	114.2	116.3		
(DF)(HF)	H-F (acceptor: HF)	0.925	0.919	0.944	0.930	0.941	0.934
	D-F (donor: DF)	0.927	0.921	0.942	0.930	0.938	0.932
	th(Db-F-H) acceptor	114.5	116.1	114.4	117.3		
	th(F-Db-F) bridge	170.0	171.2	169.5	171.4		

Table S19: Optimized single-component CCSD, multicomponent (MC) CCSD, and VPT2-CCSD bond distances (angstroms) and angles (degrees) for (HF)2 and its isotopologues, as in Table 3 of the manuscript, with the 8s8p8d8f protonic basis set (aug-cc-pVDZ, aug-cc-pVTZ electronic basis sets).

	MC CCSD	
	DZ	TZ
HCN	3.113	3.098
DCN	3.093	3.084
HNC	3.158	3.168
DNC	3.134	3.152
HCCD	0.016	0.010
HNND	0.012	0.008
HOOH	1.819	1.787
DOOH	1.813	1.783
DOOD	1.808	1.783
(HF)2	3.512	3.540
(DF)2	3.456	3.500
(HF)(DF)	3.487	3.509
(DF)(HF)	3.481	3.530

Table S20: Multicomponent CCSD dipole moments (Debye) with the 8s8p8d8f protonic basis set, as in Table 4 of the manuscript. The single-component CCSD and experimental values are in Table 4.

			CCSD	MC CCSD	VPT2-CCSD	Exp.
		DZ	43395	43216	43209	
HCN	B	TZ	44922	44730	44734	44316
		DZ	35420	35262	35301	
DCN	B	TZ	36679	36504	36559	36207
		DZ	44367	44156	44230	
HNC	B	TZ	45816	45614	45666	45332
		DZ	37292	37105	37258	
DNC	B	TZ	38477	38289	38427	38153
		DZ	34350	34107	34244	
HCCH	B	TZ	35679	35418	35501	35275
		DZ	28926	28725	28874	
HCCD	B	TZ	30043	29819	29922	29725
		DZ	24720	24553	24701	
DCCD	B	TZ	25674	25485	25591	25419

Table S21: Single-component CCSD, multicomponent (MC) CCSD, and VPT2-CCSD rotational constants (MHz) for the linear systems, as in Table 5 of the manuscript, with the 8s8p8d8f protonic basis set.

			CCSD	MC CCSD	VPT2-CCSD	Exp.
		DZ	297952	289033	294115	
HNNH	A	TZ	308695	301007	305163	299829
		DZ	38786	38627	38560	
	B	TZ	39831	39689	39608	39103
		DZ	34319	34073	33973	
	C	TZ	35279	35065	34935	34475
		DZ	218998	214065	216944	
HNND	A	TZ	227185	222854	225339	221373
		DZ	35582	35402	35367	
	B	TZ	36494	36324	36281	35854
		DZ	30609	30378	30299	
	C	TZ	31443	31233	31134	30746
		DZ	178276	175080	176909	
DNND	A	TZ	185145	182298	183929	180613
		DZ	32436	32244	32229	
	B	TZ	33231	33043	33024	32665
		DZ	27443	27229	27167	
	C	TZ	28174	27973	27898	27568

Table S22: Single-component CCSD, multicomponent (MC) CCSD, and VPT2-CCSD rotational constants (MHz) for HNNH, HNND, and DNND, as in Table 6 of the manuscript, with the 8s8p8d8f protonic basis set.

			CCSD	MC CCSD	VPT2-CCSD	Exp.
		DZ	300508	289838	297836	
HOOH	A	TZ	307416	300238	305036	301873
		DZ	26141	25981	25842	
	B	TZ	26932	26814	26660	26192
		DZ	25353	25132	24943	
	C	TZ	26124	25933	25735	25118
		DZ	211483	205429	210025	
DOOH	A	TZ	216537	212381	215257	212751
		DZ	24786	24642	24542	
	B	TZ	25506	25397	25286	24831
		DZ	23547	23357	23194	
	C	TZ	24235	24066	23899	23423
		DZ	163804	159785	162912	
DOOD	A	TZ	167817	165041	167060	165085
		DZ	23214	23094	23018	
	B	TZ	23857	23769	23682	23329
		DZ	22149	21970	21838	
	C	TZ	22776	22612	22479	22019

Table S23: Single-component CCSD, multicomponent (MC) CCSD, and VPT2-CCSD rotational constants (MHz) for HOOH, DOOH, and DOOD, as in Table 7 of the manuscript, with the 8s8p8d8f protonic basis set.
